# Seroprevalence of transfusion-transmissible infections (HBV, HCV, syphilis and HIV) among prospective blood donors in a tertiary health care facility in Calabar, Nigeria; an eleven years evaluation

**DOI:** 10.1186/s12889-018-5555-x

**Published:** 2018-05-22

**Authors:** Henshaw Uchechi Okoroiwu, Ifeyinwa Maryann Okafor, Enosakhare Aiyudubie Asemota, Dorathy Chioma Okpokam

**Affiliations:** Haematology Unit, Department of Medical Laboratory Science, University of Calabar, Calabar, Nigeria

**Keywords:** Blood donors, HBV, HCV, HIV, TTI, Blood donors in Calabar

## Abstract

**Background:**

Provision of constant and safe blood has been a public health challenge in Sub-Saharan Africa with high prevalence of transfusion-transmissible infections (TTIs). This study was aimed at determining the trend and seroprevalence of HBV, HCV, syphilis and HIV across the years within study among prospective blood donors at blood bank in University of Calabar Teaching Hospital (UCTH), Calabar, Nigeria.

**Methods:**

A retrospective analysis of blood donor data from January 2005 to December 2016 was conducted in Blood Bank/Donor Clinic of University of Calabar Teaching Hospital, Calabar, Nigeria. Sera samples were screened for hepatitis B surface antigen (HBsAg), antibodies to hepatitis C virus (HCV), human immunodeficiency virus (HIV) 1 and 2 and *Treponema pallidum* using commercially available immunochromatic based kits.

**Results:**

Out of the 24,979 screened prospective donors in the 2005–2016 study period, 3739 (14.96%) were infected with at least one infective agent. The overall prevalence of HBV, HCV, syphilis and HIV were 4.1, 3.6, 3.1 and 4.2%, respectively. During the period of study, the percentage of all transfusion-transmissible infections declined significantly with remarkable decline in HIV. The study showed male dominated donor pool (98.7%) with higher prevalence (4.2%) of transfusion-transmissible infections than in female donors (0.0%). Commercial donors constituted majority (62.0%) of the donors and as well had the highest prevalence of transfusion-transmissible infections. Majority (62.9%) of the donors were repeat donors.

**Conclusion:**

HBV, HCV, syphilis and HIV have remained a big threat to safe blood transfusion in Nigeria and Sub-Saharan Africa at large. Strict adherence to selection criteria and algorithm of donor screening are recommended.

## Background

Blood transfusion can be a lifesaving intervention. However, like all treatments, it may result in acute or delayed complications and carries the risk of transfusion-transmissible infections such as HIV, hepatitis B and C, syphilis, malaria, etc. as well as hemolysis [1, 2]. Blood safety remains a major public health problem in Sub-Saharan Africa owing to inadequacies of national blood transfusion policies and services, appropriate infrastructures, qualified personnel and financial resources [3]. Nigeria established a national blood transfusion policy through a published set of guidelines in December 2006 which gave birth to the National Blood Transfusion Service. The national blood policy is essentially made up of sets of action plans which are geared towards the provision of safe, available and affordable blood donor units. It is structured into the following strata: the national blood transfusion service (NBTS), the zonal blood service centers, state and local government areas blood service centers, the armed forces blood service centers, private and other nongovernmental health establishments [4, 5]. Despite these efforts, in Nigeria, there is still lack of political will and open-mindedness to innovative ways to improve supply and safety of blood through voluntary donors [6]. Transfusion-transmissible infections remain one of the most serious complications of blood transfusion [7]. Transfusion of infected blood is the cause of 5–10% of HIV infection in Sub-Saharan Africa [8] and 12.5% of the patients who received blood transfusions are at risk of post-transfusion hepatitis [9].

The 2015 global prevalence of HBV infection in general population as reported by World Health Organization [10] was 3.5% accounting for about 257 million persons. Prevalence was highest in Western Pacific (6.2%) and Africa (6.1%). Eastern Mediterranean region, South East Asia region, European region, and Regions of Americas had 3.3, 2.0, 1.6 and 0.7% prevalence, respectively [10]. More so, the Polaris Observatory Collaborators in a survey using 128 countries reported mean global HBV prevalence of 4.9% with China, India, Nigeria, Indonesia, and the Philippines accounting for more than 57% of all the HBsAg positive cases [11].

The 2015 global prevalence of HCV as reported by WHO estimated that 71 million persons were living with HCV infection in the world accounting for 1% of the population. Of these, the highest prevalence were recorded in the Eastern Mediterranean region (2.3%) and European region (1.5%). African region, Regions of Americas, Western Pacific region and South East Asia region had 1.0, 0.7, 0.7 and 0.5% prevalence, respectively [10].

Globally, 36.7 million people were living with HIV at the end of 2016. An estimated 0.8% of adults aged 15–45 years worldwide are living with HIV, although the burden of the epidemic continues to vary considerably between countries and regions. Sub-Saharan Africa remains the most severely affected with nearly 1 in every 25 adults (4.2%) living with HIV and accounting for nearly two-third of people living with HIV worldwide [12].

The global incidence of syphilis was 25.1 case per 100,000 adult population among the 55 countries that reported in the GARPR (Global Aids Response Progress Reports) in 2014 [13]. Each year, there are about 5.6 million people living with syphilis [14].

Only continuous and strict implementation of donor selection, screening tests and effective inactivation procedures can ensure the elimination, or at least reduction of the risk of acquiring transfusion-transmissible infections [15].

Assessment of data on the prevalence of transfusion transmissible infections among blood donors permits evaluation of the occurrence of infections in the blood donor population and consequently, the safety of the collected donations. It also gives an idea of the epidemiology of the transfusion-transmissible infections in the study community [16].

## Methods

**Fig. 1:**
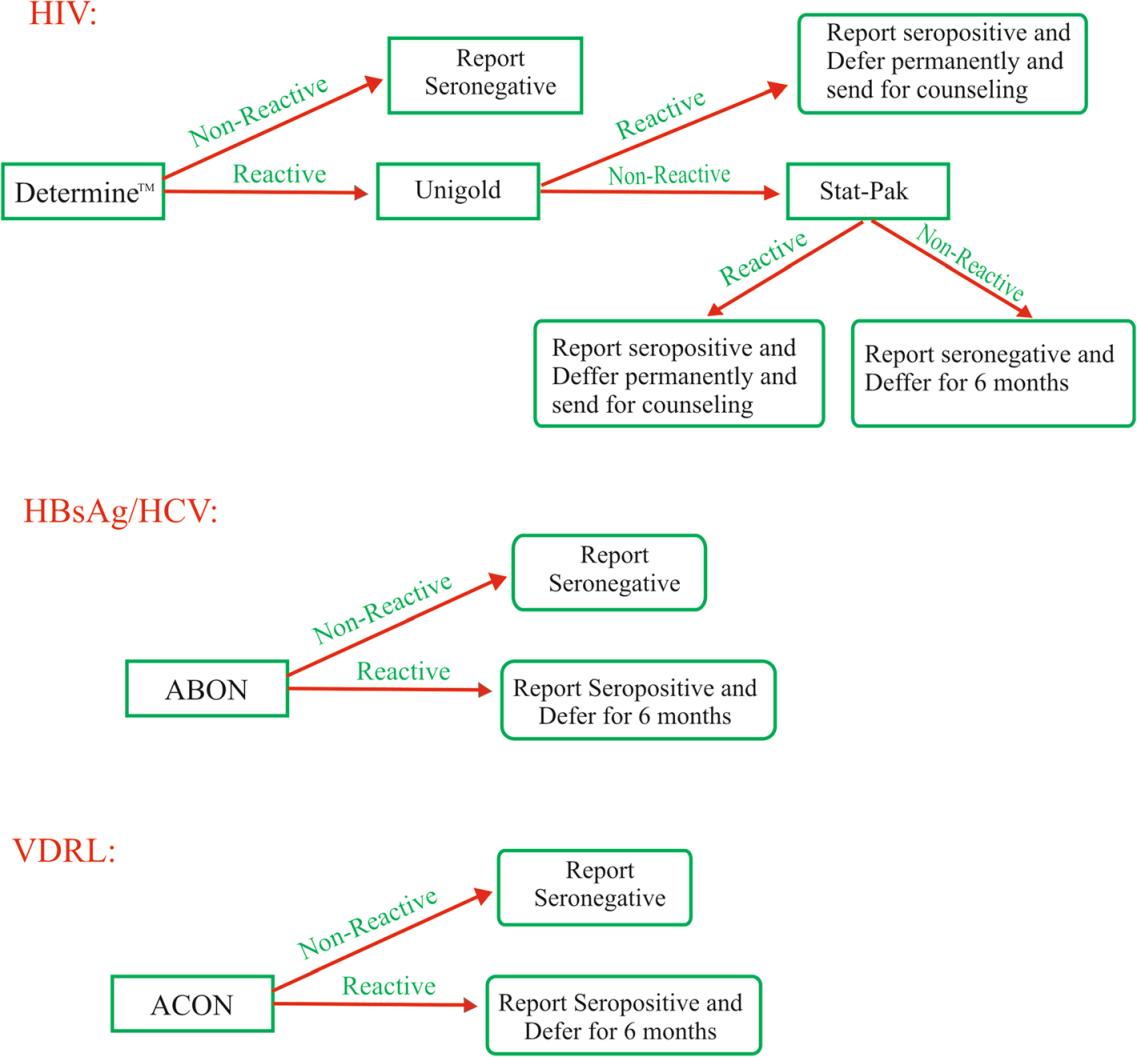
Algorithm for serological screening for blood donors

A retrospective analysis of blood donor data from January 2005 to December 2016 was conducted in Blood Bank/Donor Clinic of University of Calabar Teaching Hospital, Calabar, Nigeria. The donors were within 18–65 years of age and weighed not less than 50 kg. Within the study period 24,979 apparently healthy prospective donors were tested for HBsAg, HCV, VDRL (syphilis) and HIV. Tests were performed using commercially available kits according to the manufacturers’ instructions. HCV and HBsAg screening were done using serum/plasma rapid immunochromatographic kits developed by ABON Biopharm (Hangzhou) Co. limited. The HIV status of the donors were determined using two rapid HIV test methods; Determine^™^ HIV 1/2 (Alere Medical Co. Ltd., Japan) and Uni-Gold^™^ Recombigen® HIV 1/2 (Trinity Biotech, Ireland) test kits. Sera from all donors were tested for the presence of treponemal antibodies using ACON rapid immunochromatographic VDRL (ACON Laboratories, Inc., San Diego, CA, USA) kits following the manufacturer’s instructions. The algorithm used in the blood bank assessed is detailed in Fig. 1.

## Results

The overall number of prospective donors screened for donation within 2005–2016 period was 24,979 comprising of 24,654 (98.70%) males and 325 (1.30%) females. Of these, 1013 (4.1%) were reactive for HBsAg, 896 (3.6%) for HCV, 786 (3.6%) for syphilis and 1044 (4.2%) for HIV (Table 1). The total number of those infected with at least one agent was 3739. The percentage prevalence of transfusion transmissible infection in the study period was 14.96% while the percentage prevalence of HBV, HCV, Syphilis and HIV infected donors were 4.1, 3.6, 3.1 and 4.2%, respectively (Table 1). The year-to-year fluctuations in the overall prevalence of TTI in the prevalence of all the infections were all statistically significant (Table 1).

**Table 1:** Transfusion-transmissible infections in blood donors at University of Calabar Teaching Hospital during the period of 2005–2016

Year	No. of blood donors screened	Prevalence							
		HBV		HCV		Syphilis		HIV	
		N.	%	N.	%	N.	%	N.	%
2005	1410	79	5.6	58	4.1	54	3.8	81	5.7
2006	1871	82	4.4	108	5.8	80	4.2	103	5.5
2007	2110	94	4.5	48	2.3	76	3.6	121	5.7
2008	2118	98	4.6	52	2.5	44	2.1	80	3.7
2009	2225	115	5.1	25	1.1	83	3.7	152	6.8
2010	2327	77	3.3	61	2.6	83	3.6	124	5.3
2011	3234	121	3.7	272	8.4	183	5.7	118	3.6
2012	2008	81	4.0	67	3.3	78	3.9	59	2.9
2013	1611	60	3.7	30	1.9	15	0.9	96	6.0
2014	1883	77	4.1	79	4.2	43	2.3	57	3.0
2015	2178	60	2.8	39	1.8	9	0.4	18	0.8
2016	2004	69	3.4	57	2.8	38	19	35	1.7
Total	24,979	1013	4.1	896	3.6	786	3.6	1044	4.2
*P* value		X^2^(11) *N* = 32.153, *P* = 0.001		X^2^(11) = 316.068, *P* < 0.01		X^2^(11) *N* = 171.003, *P* < 0.01		X^2^(11) *N* = 182.261, *P* < 0.01	
Gender									
Male	24,654 (98.70)	1012	4.1	896	3.6	786	3.2	1044	4.2
Female	325 (1.30)	1	0.3	0	0	0	0	0	0

*N* absolute number of positive blood donors, % percentage of positive blood donors, *P* significance of year-to-year fluctuations, determined by chi square test, *HBV* Hepatitis B virus, *HCV* Hepatitis C virus, *HIV* Human Immunodeficiency Virus

Within the study period, a declining trend in prevalence of TTI-positivity among donors was observed which was the result of decreasing number of HBV, HCV, syphilis and HIV positive donors (Table 1).

**Fig. 2:**
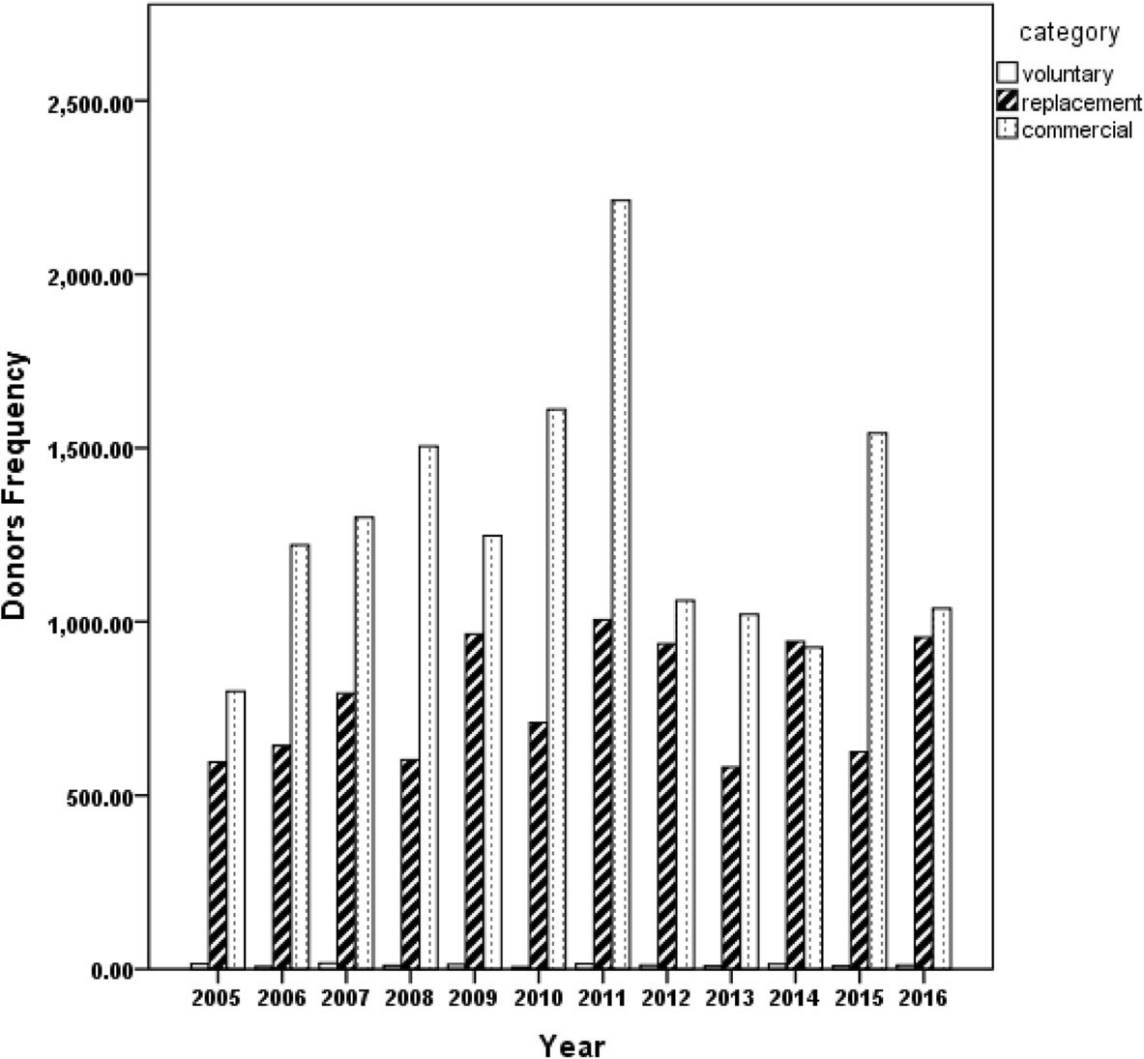
Blood donation pattern over the period of 2005–2016 in University of Calabar Teaching Hospital, Nigeria

There were 137 (0.5%) voluntary, 9355 (37.5%) family replacement and 15,487 (62.0%) commercial donors in this study. There was no significant difference in the trend of donors over the years (Fig. 2). Transfusion transmissible infection was observed in 3 (2.2%) of the voluntary donors; consisting of 2 (1.5%), 1 (0.7%) and 0 (0%) HBsAg, HCV, Syphilis and HIV reactive cases, respectively. The family replacement donors had 1160 (12.4%) TTIs frequency consisting of 303 (3.2%), 253 (2.7%) and 387 (4.1%) reactive cases of HBsAg, HCV, Syphilis and HIV, respectively. More so, 15,487 (62.9%) cases of TTIs consisting of 708 (4.6%), 642 (4.1%), 569 (3.7%) and 657 (4.2%) cases of HBsAg, HCV, Syphilis and HIV, respectively were observed in the commercial donors (Table 2). There were 9267 (37.1%) first time and 15,712 (62.9%) repeat donors in this study (Table 3).

**Table 2:** Prevlence of Transfusion-transmissible infections in the three blood donor categories

Donor Category	Total Number of Samples (%)	Number of Positive Cases (%)				Total TTI (%)
		HBV (%)	HCV (%)	Syphilis (%)	HIV (%)	
Voluntary Donors	137 (0.5)	2 (1.5)	1 (0.7)	0 (0.00)	0 (0.00)	3 (2.2)
Replacement Donors	9355 (37.5)	303 (3.2)	253 (2.7)	217 (2.3)	387 (4.1)	1160 (12.4)
Commercial Donors	15,487 (62.0)	708 (4.6)	642 (4.1)	569 (3.7)	657 (4.2)	15,487 (62.9)
*P* value		X^2^(2) =743.4	X^2^(2) =698.3	X^2^(2) =157.6	X^2^(2) =69.8	X^2^(2) = 26,808
		*P*< 0.01	*P*< 0.01	*P*< 0.01	*P*< 0.01	*P*< 0.01

**Table 3:** Categorization of blood donors based on frequency of blood donation

Donor Category	Frequency	%
First time	9267	37.1
Repeat	15,712	62.9

## Discussion

This study recorded the overall prevalence of various transfusion-transmissible infections in prospective blood donors at the blood bank of University of Calabar Teaching Hospital, Nigeria in the period from 2005 to 2016. Within the study period, we found that 14.96% (*n* = 3739) of the prospective donors were positive for transfusion-transmissible infections (TTIs). The decreasing trend in the prevalence of TTI observed in this study is in line with the global trend resulting from improved control of sexually transmitted infections [17], introduction of mandatory screening for TTI and launching of intervention programmes [18, 19]. Our finding is lower than another study in Nigeria [20] and Burkina Faso [21] that had 19.3 and 19.3%, respectively, but higher than earlier report from Ethiopia [22] with 11.5%. The difference in the prevalence may be due to differences in health care system in the different study settings as well as varying magnitude of risk factors for contracting transfusion transmissible infections [22] in the various settings.

In this study the prevalence of HBV was 4.1%. This value is lower than 14.3, 11.1, 26.0 and 20.3% reported in Jos [23], Kano [20], Taraba [24] and Ibadan [25], respectively in similar studies within Nigeria. However, lower value (1.67%) has been reported in Portharcourt [26], Nigeria. Similar findings have been reported in other Sub-Sahara African countries. The prevalence of HBV found in this study in Calabar Nigeria is similar to 4.7% reported in Ethiopia [22], while same is lower than 11.59, 7.51, 11.2 and 14.96% reported in Ghana [27], Benin [28], Cameroon [29] and Burkina Faso [30], respectively. Prevalence of hepatitis B observed in this study could be ranked low when compared with other Sub Sahara African countries and regions in Nigeria.

The prevalence of HCV in this study was 3.6%. This value is similar to 3.4 and 4.1% reported in Kano [30] and Benue [31], respectively. However, this finding is higher than 0.5 and 2.0% reported in Portharcourt [32] and Nnewi [33], respectively but lower than 6.1 and 6.0% reported in Jos [34] and Oshogbo [35] respectively, being studies within Nigeria. Comparatively with some Sub-Saharan regions, similar values; 3.2 and 4.4% have been reported in Kenya [36] and Ghana [37], respectively. However, lower value (0.4%) have been reported in Ethiopia [22] whereas higher value (8.6%) has been reported in Burkina Faso [21]. The reason for the relatively lower rate of seroprevalence of HBV and HCV in this study compared with other studies is unclear, but could be ascribed to differences in methodology, sensitivity and specificity of the various rapid test kits used in the different studies. The improvement in technology might make current screening reagents to be more specific and reliable, and could also be a pointer that there are geographical differences in prevalence.

The overall prevalence of syphilis in this study was 3.1%. The value obtained from this study is similar to 3.6 and 2.61% reported in Maiduguri [38] and Ile Ife [39], respectively within Nigeria. However, lower value of 0.1% has been reported in Portharcourt [26]. Similar result has been reported from Sub-Saharan region such as Burkina Faso [21] (3.96%). However, lower values have been reported in Ethiopia [22] (0.1%) and Kenya [36] (1.2%) whereas a higher value of 7.5% has been reported in Ghana [40]. The reason for the disparity in the prevalence of syphilis observed in this study when compared to other studies may be due to geographical differences in the prevalence of syphilis as well as methodological differences. It has been earlier reported that *T. pallidum* particle agglutination assay are more sensitive than rapid plasma reagin and *T. pallidum* hemagglutination assay [3].

Seroprevalence of HIV in this study was 4.2%. This prevalence is similar to 4.6 and 3.8% reported in similar Nigerian studies in Sokoto [41] and Kano [42]. However, lower values of 1 and 2.8% have been reported in Portharcourt [26] and Kaduna [43]. Data from some other Sub-Saharan region has reported similar result in Ghana [37] (4.9%). However, lower values of 0.1 and 2.21% have been reported in Ethiopia [22] and Burkina faso [21], respectively. Though the overall HIV prevalence in this study is high, there was a sharp decline in the prevalence across the years from a score of 5.7% in the starting year to 1.7% in the ending year. The earlier rise in HIV seroprevalence observed in this study corresponds to the peak of HIV epidemic at the era when HIV was not recognized as public health concern. This was also when people were in denial of the disease. The subsequent decline that ensued could be interpreted as the outcome of the various intervention programmes that have been implemented over the years.

This study observed significantly declining trend of transfusion-transmissible infections within the period under study. This observation is in consonance with reported decline in HIV seroprevalence in Nigeria [26, 42]. This trend gives credence to much of the HIV/AIDS intervention programmes launched over the years.

There was a male dominated donor pool (98.70%) in this study. This demographic pattern have been replicated in earlier studies from other parts of Nigeria [44, 45]. Similar trend has been reported in India (95.20%) [46], Pakistan (99.62%) [47], Cameroon (82.0%) [48], Ethiopia (86.8%) [49] and Mexico (81.86%) [50]. This observation could be attributed to the cultural dogma that women should abstain from blood donation as they do lose blood monthly via menstruation. More so, the present study and the studies with similar trends were comprised mostly of replacement and remunerated donors. Male donors have been reported to be motivated by altruism and remuneration benefits [51]. However, this observation was at variance with reports from United States of America (51.7%) [52], Spain (54.0%), Portugal (57.0%), Belgium (54.6%), Netherlands (50.0%,), Denmark (50.0%), France (50.0%), United Kingdom (47.0%) and Finland (45.0%) [51] where the values were almost at par in both sexes. This non gender difference may be attributed to the fact that majority of the blood donations in these developed countries were voluntary donations which is mainly driven by altruism which is shared by both sexes [51]. Females have been reported to be motivated to donate mainly by altruism rather than males who do for both altruism and remuneration benefits [51]. Male donors recorded the highest prevalence of TTIs. This finding is similar to the report of Okocha et al. [44]. Zaheer et al. [47] attributed this to the view that women are confined to home settings, and therefore, are comparatively less exposed to the risks factors associated with transfusion transmissible infections as compared to males.

This study identified commercial donors as the predominant donor type, followed by the family replacement donors, while the voluntary donors were the least in number. The amount of voluntarily donated blood has continued to fall over the years in Nigeria due to logistics and organizational problems associated with the national blood transfusion service. The net result is that commercial blood donation becomes the order of the day. This finding is in consonance with previous reports [35, 44]. However, the finding is at variance with previous report by Damulak et al. [53] in Jos. This disparity could be attributed to the fact that the study in Jos was carried out in the National Blood Transfusion Service Center whereas our study was done in a hospital based blood bank. Blood drive for voluntary blood donation is a regular practice in national blood transfusion centers in Nigeria unlike hospital based blood banks that rely mostly on walk in voluntary donors. Commercially remunerated blood donors recorded the highest prevalence of TTIs, while voluntary donors had the least. This finding further corroborates the earlier suggestion by World Health Organization (WHO) that voluntary donors are less likely to transmit transfusion transmissible-infection than family replacement and commercial donors [54]. An individual in desperate financial need is less likely to reveal his/her actual health status and the monetary reward could make it appealing to high risk donors.

A larger part (62.9%) of donors in this study were repeat donors. This is owing to the fact that majority of the donors were commercial donors. Commercial donors are more likely to repeat because of the financial remuneration that is intended to motivate it.

### Limitations of study

The rapid kits used for this study are in vitro qualitative methods. The quantitative values cannot be determined by these screening tests. A negative result at any time does not preclude the possibility of latent infection as most of the test kits were antibody based (HCV, HIV and syphilis) and surface antigen based (HBV). The essence of the pre-donation screening is to determine deferment status; hence, the algorithm is direct except for HIV that has three steps. However for logistics reasons, all HIV screening in this study were benched at UNIGOLD (second stage). More so, the borderline between family replacement and commercial donors is gray as sometimes patient relatives may recruit commercial donors and present same as replacement on their own.

## Conclusion

The present study clearly showed a high prevalence of transfusion-transmissible infection among blood donors in the studied population. The high remunerated blood donor pool and male dominated donor population call for renewed attention to donor recruitment in blood transfusion centers in the country and also consistent enlightenment programmes on transfusion-transmissible infections with a view of reversing this trend. There is a need for stringent selection of blood donors with the emphasis on getting voluntary donations and comprehensive screening of donors for TTIs.

## Abbreviations

HBsAg: Hepatitis B surface antigen
HBV: Hepatitis B virus
HCV: Hepatitis C virus
HIV: Human immunodeficiency virus
TTIs: Transfusion-transmissible infections
VDRL: Venereal disease research laboratory
